# Chiropractic attitude and utilisation of evidence-based practice in South Africa: a secondary analysis

**DOI:** 10.1186/s12998-024-00534-3

**Published:** 2024-04-30

**Authors:** Sharné Naidoo, Nicole Karensa Hoenselaar, Christopher Yelverton

**Affiliations:** Department of Chiropractic, University of Johannesburg, Beit Street, 2028 Johannesburg, Gauteng, South Africa

**Keywords:** Evidence-based practice, Chiropractic, Cross-sectional studies, Survey

## Abstract

**Background:**

Evidence-Based Practice (EBP) is a model utilised by the majority of healthcare professionals and is a clinical framework that assists with decision-making related to patient care, to improve outcomes and patient satisfaction. The study aimed to analyse the attitudes, skills, and utilization of evidence-based practice (EBP) among South African chiropractors, focusing on perceived skill levels, training, use and identifying facilitators and barriers to EBP application.

**Methods:**

A descriptive cross-sectional quantitative secondary analysis was conducted by inviting registered chiropractors in South Africa (*n* = 920) to participate in an anonymous online questionnaire using the Evidence-Based Practice Attitude and Utilisation Survey (EBASE).

**Results:**

A total of 132 chiropractors completed the survey, yielding a response rate of 14.4%. Of the respondents, 59.9% were female, 52.3% were between 26 and 35 years old, and 63.3% had graduated from the University of Johannesburg. A third of respondents stated they have poor clinical research skills. Over half of the respondents (56.8%) indicated that EBP constituted a significant part of their education except for completing systematic reviews or meta-analyses. Published clinical evidence was ranked 6th as a source of information for clinical decisions. The obstacles indicated were time constraints and a lack of clinical research in complementary and alternative medicine. Access to the internet, databases and research tools were facilitators that were deemed to be “very useful” in promoting EBP.

**Conclusion:**

The majority of South African chiropractors are generally favourable towards EBP, and this practice therefore appears to be utilised and embraced, with the requisite skills.

**Supplementary Information:**

The online version contains supplementary material available at 10.1186/s12998-024-00534-3.

## Introduction

Chiropractic is among the most widely utilised complementary and alternative medicine (CAM) therapies [1–4]. In South Africa, chiropractic was first introduced into the country in the 1920s and has now been established as a profession with over 900 practitioners [5]. Chiropractors in South Africa play an active role in the healthcare system as primary care providers for musculoskeletal disorders [6–8]. Chiropractic involves a wide range of manual techniques and electrotherapies, such as chiropractic manipulation, myofascial pain therapy and shockwave therapy [9]. Chiropractors can obtain these specialized skills through two institutions in South Africa. These institutions offer a master’s qualification required by the regulatory council, ensuring practitioners adopt an evidence-based approach in their practice [7].

Evidence-based practice (EBP) is a clinical framework which has gained prominence in the healthcare system, including the chiropractic profession [10]. EBP has been implemented since the 1990s, to assist healthcare professionals in achieving better patient outcomes that take into consideration patient values, recent literature, and the practitioner’s clinical experience [11]. This approach aligns with the increasing understanding towards patient-centred care, which emphasises the importance of achieving satisfactory treatment outcomes as a foundation in healthcare [10]. EBP impacts patient care and may also direct healthcare education, policies, reimbursement, and clinical management [12].

There is growing recognition from healthcare providers that clinical research needs to be more patient-orientated and practically applicable to clinical practice [13]. To assist practitioners in making informed decisions about patient management and treatment, Clinical Practice Guidelines (CPGs) are developed. It is important for EBP to align with these guidelines, as it can help optimize patient outcomes, potentially leading to cost reduction and minimizing unnecessary treatments [8].

The efficacy of treatments in practice is often underestimated, despite research findings showing a significant gap [8]. EBP can be divided into research utilization (RU) and knowledge translation (KT). RU refers to research-based knowledge applied in practice, whilst KT enhances health outcomes through knowledge gained from research findings [8]. To enhance treatment effectiveness and efficiency, healthcare providers, like chiropractors, should prioritize strategies to increase RU and promote KT in clinical practice in an attempt to bridge the research gap [8].

Chiropractors, in justifying their profession to other healthcare providers and the general public, have reported that the profession has limited experimentally based research programmes focusing specifically on chiropractic [14]. Peer conversations were used to resolve clinical concerns faced in practice rather than referring to literature and developing their own clinical experience [14]. However, the general trend appears to be that there is an increased acceptance of EBP in chiropractic, with research directed towards more practice-or patient-relevant areas to enhance and improve diagnosis and treatment outcomes for musculoskeletal disorders [15]. EBP can additionally enhance the integrative collaboration between allopathic and CAM practitioners by emphasizing a multidisciplinary approach for improved condition prognosis [15].

Several studies have been conducted in Australia, Europe, North America, and South America [8] regarding the attitudes, beliefs, and utilisation of EBP within the chiropractic profession. The findings have indicated a positive outlook on EBP; however, there was a lack of uptake and practice of the available relevant research findings. Gaining an African perspective can assist in adding to the global overview of chiropractors’ attitudes and uptake of EBP. The information gained from this study could also identify key strengths and weaknesses to enhance the uptake of EBP in South African chiropractors. The aim of this study was to examine the attitudes, skills, and utilisation of evidence-based practice (EBP) among South African chiropractors. The study specifically aimed to address the following objectives: (a) attitudes towards EBP, (b) perceived skill levels in EBP, (c) EBP training and education, (d) use of evidence-based practice, and (e) identify facilitators and barriers to EBP adoption.

## Methods

### Design

The study design utilised a quantitative descriptive secondary analysis using data from four primary studies from the University of Johannesburg. The four primary studies collectively aimed to determine the attitudes and utilisation of EBP among chiropractors in the nine provinces within South Africa; with each study collecting data from one or more provinces. These primary studies were active for 90 days (from the 27th of May 2021 to the 25th of August 2021) and were distributed as a survey link via the Allied Health Professions Council of South Africa (AHPCSA) and the Chiropractic Association of South Africa (CASA) databases to registered chiropractors in South Africa.

### Sample and setting

The survey utilized QuestionPro (QuestionPro, Austin, Texas, USA), an online software, to collect responses including an electronic information letter and consent form. This information letter stated the study’s objectives, the primary researchers’ contact details, and confidentiality of the data. Respondents had to complete a consent form to confirm their participation. The anonymous nature of the questionnaire allowed respondents to withdraw from the study before submitting the questionnaire.

At the time of this study, the sample population consisted of 920 registered chiropractors in South Africa and used purposive sampling (L. Mullinder, personal communication, May 27, 2021). The study included chiropractors registered with the AHPCSA, South Africa’s regulatory body for chiropractic. The AHPCSA and CASA sent an email to invite chiropractors to complete the survey using the QuestionPro link, as all registered chiropractors provided their contact information. A minimum of 130 responses was required for the data to be statistically significant, which is a minimum of 14% (confidence level of 95% with a margin of error of 8%) of the total population size, according to the statistician assigned to this study. Chiropractors who were registered with the AHPCSA and not primarily practicing in one of the nine South African provinces were excluded from the study.

### Measurement

The original EBASE questionnaire was established by Leach and Gillham [16]. Alcantara and Leach adapted the Evidence-Based Practice Attitude and Utilisation Survey (EBASE), which was used to analyze and interpret the attitudes and utilisation of EBP among practitioners [15]. The survey consists of seven parts, A– G. Part A was for the collection of demographic information relating to respondents’ age, education, and sex and was the only part to be adapted for the socio-demographic particulars from a South African perspective. Part B investigates chiropractors’ attitudes towards evidence-based practice, where a 5-point Likert scale is used and scaled from 1 (strongly disagree) to 5 (strongly agree). The skills of chiropractors regarding evidence-based practice are identified in Part C using a 5-point Likert scale ranging from 1 (poor) to 5 (advanced). Part D rates the knowledge of chiropractors in the five areas of evidence-based practice. For each area, respondents indicate the highest level of training/education which includes no training, formal education, short courses, informal studying and an additional option should none of the listed items be applicable. The utilisation of evidence-based practice by chiropractors is rated in Part E. The first subsection investigates the frequency of research activities using a 5-point Likert scale to categorise the responses ranging from never to 16 + times per month. The following subsection estimates the percentage that chiropractors base their practice on clinical research using six categorical responses ranging from 0 to 100%. The last subsection of Part E ranks ten sources of information, from 1 (most frequently used) to 10 (least frequently used), and how these sources influence their clinical decision-making in practice. Part F investigates the potential barriers to evidence-based practice by chiropractors using a 4-point ranging from “1 - not a barrier” to “4 - a major barrier”. Part G assesses the role of facilitators in chiropractors’ adoption of evidence-based practice on a 4-point scale ranging from “1 - not useful” to “4 - very useful.”

The original survey’s content validity was confirmed, along with a good internal reliability of 0.87 using Cronbach’s alpha. The EBASE tool was completed twice, demonstrating strong test-retest reliability and moderate agreement in Part C of the questionnaire [16].

### Data analysis

The data was collected and processed through the IBM SPSS programme for statistical analysis with the assistance of Statistical Consultation Services (STATKON) at the University of Johannesburg. No missing data was reported. Frequencies and percentages were used to characterize categorical data. For continuous variables, descriptive statistics was employed to provide a basic statistical overview.

## Results

### Response rate

A total of 14.4% (*n* = 132) response rate was recorded among the 920 chiropractors registered with the AHPCSA in South Africa.

### Demographic data

This study found that 52.3% of respondents were aged 26–35, with 59.8% being female. The majority were South African graduates (97.7%), and 63.6% graduated from the University of Johannesburg. Approximately half of the respondents (52.3%) obtained their qualification between 2010 and 2019. Most respondents did not have an additional qualification after obtaining their primary chiropractic degree (60.6%) (Table 1).

**Table 1:** Demographic characteristics of respondents (*n* = 132)

Demographic Category	Variable	Number of Respondents N (%)
Age	25 and younger	10 (7.6)
	26–35 years old	**69 (52.3)**
	36–45 years old	33 (25.0)
	46–55 years old	16 (12.1)
	Older than 55	4 (3.0)
Sex	Male	53 (40.2)
	Female	**79 (59.8)**
University of graduation	University of Johannesburg	**84 (63.6)**
	Durban University of Technology	32 (24.2)
	Technikon Witwatersrand	6 (4.5)
	Technikon Natal	7 (5.3)
	Canadian Memorial Chiropractic College	1 (0.8)
	Palmer College of Chiropractic	2 (1.6)
Year of graduation	1960–1969	1 (0.8)
	1970–1979	1 (0.8)
	1980–1989	1 (0.8)
	1990–1999	9 (6.8)
	2000–2009	33 (25.0)
	2010–2019	**69 (52.3)**
	2020+	19 (14.4)
Highest level of qualification besides MHSc*/MTech^†^ degree in Chiropractic	No Other Degree	**80 (60.6)**
	Bachelor’s Degree	11 (8.3)
	Honour’s Degree	3 (2.3)
	Master’s Degree	38 (28.8)
Province of primary practice	Gauteng	**86 (65.2)**
	Kwa-Zulu Natal	19 (14.4)
	Mpumalanga	7 (5.3)
	Limpopo	2 (1.5)
	Free State	3 (2.3)
	Western Cape	15 (11.4)
Geographic setting of practice	Urban	59 (44.7)
	Suburban	**69 (52.3)**
	Rural	4 (3.0)
Role in clinical setting of practice	Associate Employee	30 (22.7)
	Sole Proprietor/Solo Practice	**74 (56.1)**
	Sole Proprietor within a group	16 (12.1)
	Owner/ Partner muti-disciplinary practice	9 (6.8)
	Partnership within group	3 (2.3)
Average number of patients seen personally (not in clinic) weekly	10 or less	19 (14.4)
	11–20	**28 (21.2)**
	21–30	**28 (21.2)**
	31–40	20 (15.2)
	41–50	14 (10.6)
	51–60	7 (5.3)
	61–70	5 (3.8)
	71–80	1 (0.8)
	81–90	2 (1.5)
	91–100	6 (4.5)
	More than 100	2 (1.5)
Main focus of chiropractic care	Paediatrics	2 (1.5)
	Family Care	46 (34.8)
	Wellness/Prevention	6 (4.5)
	Sports	9 (6.8)
	Spine	1 (0.8)
	General Musculoskeletal Care	**68 (51.5)**
Organisational status	No membership	26 (19.7)
	CASA^‡^ Member	**106 (80.3)**

Bold values indicate the highest number of responses for each item

* Master of Health Science

^†^ Master of Technology

^‡^ Chiropractic Association of South Africa

The majority of respondents practiced in Gauteng (65.2%) and suburban areas (52.3%), primarily focusing on general musculoskeletal care (51.5%). They averaged 11–20 or 21–30 patients per week (21.2%), with 56.1% of practitioners being solo practitioners. 80.3% of respondents subscribed to the Chiropractic Association of South Africa (CASA) (Table 1).

### Chiropractic attitudes towards evidence-based practice

The majority of respondents (85.6%) had a positive attitude towards the need for EBP in practice. Nearly half of the respondents (47.0%) expressed an interest in learning and enhancing new or existing skills to incorporate current outcomes into their practices. Respondents agreed (87.9%) that EBP aids in their decision-making, and improve the quality of their patients’ care (87.9%). There was a disagreement among respondents (61.3%) that applying the EBP model would place a high demand on their practice (Table 2).

**Table 2:** Chiropractic attitudes towards evidence-based practice (*n* = 132)

	Strongly Disagree N (%)	Disagree N (%)	Neutral N (%)	Agree N (%)	Strongly Agree N (%)
Evidence-based practice is necessary in the practice of Chiropractic	14 (10.6)	1 (0.8)	4 (3.0)	44 (33.3)	**69** **(52.3)**
Professional literature (journals & textbooks) and research findings are useful in my day-to-day practice	3 (2.3)	3 (2.3)	15 (11.4)	**63 (47.7)**	48 (36.4)
I am interested in learning or improving the skills necessary to incorporate evidence-based practice into my practice	3 (2.3)	3 (2.3)	11 (8.3)	53 (40.2)	**62** **(47.0)**
Evidence-based practice improves the quality of my patients’ care	3 (2.3)	3 (2.3)	10 (7.6)	52 (39.4)	**64** **(48.5)**
Evidence-based practice assists me in making decision about patient care	3 (2.3)	2 (1.5)	11 (8.3)	57 (43.2)	**59** **(44.7)**
Evidence-based practice takes into account my clinical experience when making clinical decisions	4 (3.0)	10 (7.6)	16 (12.1)	**56 (42.4)**	46 (34.8)
Evidence-based practice takes into account patient preference for treatment	5 (3.8)	31 (23.5)	33 (25)	**39 (29.5)**	24 (18.2)
The adoption of evidence-based practice places an unreasonable demand on my practice	32 (24.2)	**49** **(37.1)**	36 (27.3)	12 (9.1)	3 (2.3)

Bold values indicate the highest number of responses for each item

### Chiropractic skills towards evidence-based practice

This study found that respondents reported average skills in locating professional literature (33.3%), searching online databases (34.8%), and retrieving evidence (41.7%). Detecting knowledge gaps in practice (56.8%), using clinical research results (41.7%) and recognizing answerable clinical questions (65.9%) were reported as above average skills among respondents. Conducting clinical research was deemed to have the lowest degree of competence (33.3%) (Table 3).

**Table 3:** Chiropractic perceptions of their skills in relation to evidence-based practice (*n* = 132)

	Poor Skills N (%)	Below Average Skills N (%)	Average Skills N (%)	Above Average Skills N (%)	Advanced Skills N (%)
Identifying knowledge gaps in practice	1 (0.8)	4 (3.0)	43 (32.6)	**75** **(56.8)**	9 (6.8)
Identifying answerable clinical questions	1 (0.8)	3 (2.3)	29 (22.0)	**87** **(65.9)**	12 (9.1)
Locating professional literature	4 (3.0)	27 (20.5)	**44** **(33.3)**	38 (28.8)	19 (14.4)
Online database searching	7 (5.3)	25 (18.9)	**46** **(34.8)**	38 (28.0)	17 (12.9)
Retrieving evidence	5 (3.8)	18 (13.6)	**55** **(41.7)**	38 (28.8)	16 (12.1)
Critical appraisal of evidence	3 (2.3)	18 (13.6)	**58** **(43.9)**	47 (35.6)	6 (4.5)
Synthesis of research evidence	9 (6.8)	25 (18.9)	**61** **(46.2)**	31 (23.5)	6 (4.5)
Applying research evidence to patient cases	2 (1.5)	4 (3.0)	40 (30.3)	**70** **(53.0)**	16 (12.1)
Sharing evidence with colleagues	6 (4.5)	24 (18.2)	35 (26.5)	**53** **(40.2)**	14 (10.6)
Conducting clinical research	**44** **(33.3)**	36 (27.3)	31 (23.5)	17 (12.9)	4 (3.0)
Using findings from clinical research	4 (3.0)	18 (13.6)	43 (32.6)	**55** **(41.7)**	12 (9.1)
Conducting systematic reviews	35 (26.5)	37 (28.0)	**43** **(32.6)**	16 (12.1)	1 (0.8)
Using findings from systematic reviews	9 (6.8)	29 (22.0)	40 (30.3)	**46** **(34.8)**	8 (6.1)

Bold values indicate the highest number of responses for each item

### Evidence-based practice training and education

Table 4 shows where respondents learned about EBP, and conducting systematic reviews or meta-analyses was seen as a minor component of chiropractic education (28.0%). Evidence-based practice (56.8%), applying research evidence to clinical practice (43.9%), doing clinical research (41.7%), and critical thinking (44.7%) were identified as key components of the chiropractic curriculum.

**Table 4:** Evidence-based practice training and education (*n* = 132)

	None N (%)	Minor part of chiropractic education N (%)	Major part of chiropractic education N (%)	Seminar (< 1 day) N (%)	Short course (< 1 week) N (%)	Specific course (> 1 week) N (%)	Formal postgraduate training N (%)	Informal personal study N (%)	Other N (%)
Evidence-based clinical practice/ Evidence-based chiropractic	2 (1.5)	20 (15.2)	**75** **(56.8)**	4 (3.0)	6 (4.5)	6 (4.5)	7 (5.3)	12 (9.1)	0 (0.0)
Applying research evidence to clinical practice	7 (5.3)	33 (25.0)	**58** **(43.9)**	10 (7.6)	6 (4.5)	3 (2.3)	6 (4.5)	9 (6.8)	0 (0.0)
Conducting clinical research	16 (12.1)	36 (7.3)	**55** **(41.7)**	5 (3.8)	6 (4.5)	1 (0.8)	10 (7.6)	3 (2.3)	0 (0.0)
Conducting systematic reviews or meta-analysis	34 (25.8)	**37** **(28.0)**	31 (23.5)	11 (8.3)	2 (1.5)	1 (0.8)	8 (6.1)	8 (6.1)	0 (0.0)
Critical thinking/ critical analysis	13 (9.8)	26 (19.7)	**59** **(44.7)**	9 (6.8)	5 (3.8)	2 (1.5)	7 (5.3)	10 (7.6)	1 (0.8)

Bold values indicate the highest number of responses for each item

### Chiropractic utilisation of evidence-based practice

Table 5 reveals that 61.4% of respondents’ practices is either moderately, largely or completely reliant on clinical research findings. However, 38.6% of respondents’ practices are based on less than 50% of clinical research evidence.

**Table 5:** Percentage of respondents’ practice based on clinical research evidence (*n* = 132)

Category	Number of respondents N (%)
None (0%)	2 (1.5)
Very small proportion (1–25%)	23 (17.4)
Small proportion (26–50%)	26 (19.7)
Moderate (51–75%)	**47 (35.6)**
Large proportion (76–99%)	29 (22.0)
All (100%)	5 (3.8)

Bold values indicate the highest number of responses for each item

### Sources of information for evidence-based practice

The data ranked information sources based on their frequency of usage by respondents. Chiropractors’ personal intuition was the most frequently used source of information (SD = 4.6). Consultation with the literature, such as CPGs (SD = 4.9), textbooks (SD = 5.2) and published clinical evidence (SD = 5.3) were moderately used. The least frequently used source was published experiments (SD = 6.9). Practitioners did not often use patient preference (SD = 6.1) or consultations with other colleagues or experts (SD = 5.0) for clinical decision-making (Table 6).

**Table 6:** Sources of information used by chiropractors for clinical decisions (*n* = 132)

	Mean SD (%)	Rank
Personal Intuition	4.6 (2.4)	1
Clinical Practice Guidelines	4.9 (2.7)	2
Traditional Knowledge	4.9 (3.0)	3
Consulting Practitioners or experts	5.0 (2.3)	4
Textbooks	5.2 (2.5)	5
Published Clinical Evidence (i.e., clinical trials)	5.3 (3.3)	6
Personal Preference	5.7 (2.9)	7
Patient Preference	6.1 (2.8)	8
Trial and Error	6.5 (2.6)	9
Published experimental/laboratory evidence (i.e., animals or test tube studies)	6.9 (3.3)	10

### Chiropractic barriers to the uptake of evidence-based practice

The most common barrier to using EBP was a lack of time (56.8%) even though 61.4% of respondents had access to resources such as a computer or online databases. The majority of respondents (59.8%) reported a willingness to be interested in EBP even if it was irrelevant to Chiropractic (51.5%), but a sizable number (47.0%) cited a lack of incentive for EBP. Insufficient abilities in critically evaluating research (43.2%) or implementing research results in a clinical environment (50.8%) are factors that may restrict the utilisation of EBP in a chiropractor’s practice (Table 7).

**Table 7:** Chiropractic barriers to the uptake of evidence-based practice (*n* = 132)

	Not a Barrier N (%)	Minor Barrier N (%)	Moderate Barrier N (%)	Major Barrier N (%)
Lack of Time	27 (20.5)	30 (22.7)	**46 (34.8)**	29 (22.0)
Lack of Resources (i.e., access to a computer, the internet or online databases)	**81 (61.4)**	30 (22.7)	13 (9.8)	8 (6.1)
Lack of clinical evidence in complementary and alternative medicine	32 (24.2)	**48 (36.4)**	42 (31.8)	10 (7.6)
Insufficient skills for locating research	**54 (40.9)**	44 (33.3)	27 (20.5)	7 (5.3)
Insufficient skills for interpreting research	**52 (39.4)**	50 (37.9)	26 (19.7)	4 (3.0)
Insufficient skills to critically appraise/evaluate research	40 (30.3)	**57 (43.2)**	29 (22.0)	6 (4.5)
Insufficient skills to apply research finding to clinical practice	50 (37.9)	**67 (50.8)**	11 (8.3)	4 (3.0)
Lack of incentive to participate in evidence-based practice	**62 (47.0)**	36 (27.3)	29 (22.0)	5 (3.8)
Lack of interest in evidence-based practice	**79 (59.8)**	36 (27.3)	15 (11.4)	2 (1.5)
Lack of relevance to Chiropractic	**68 (51.5)**	38 (28.8)	19 (14.4)	7 (5.3)
Lack of university support for evidence-based practice	**56 (42.7)**	36 (27.3)	23 (17.4)	17 (12.9)
Lack of industry support for evidence-based practice	**48 (36.4)**	39 (29.5)	29 (22.0)	16 (12.1)
Patient preference for treatment	39 (29.5)	**56 (42.4)**	29 (22.0)	8 (6.1)

Bold values indicate the highest number of responses for each item

### Chiropractic facilitators to the uptake of evidence-based practice

Respondents stated that all information sources were extremely helpful in allowing the application of EBP (Table 8). The capacity to access full-text journals (76.5%) was the most important facilitator of increased practitioner research intake, followed by access to the internet and free access to databases that normally need licensing payments (72.7%). Access to research rating tools that aid with the critical appraisal of one study (47.7%) or online resources that assist the practitioner with their own critical evaluation of several studies (46.2%) were the least beneficial tools utilized by practitioners.

**Table 8:** Chiropractic facilitators to the uptake of evidence-based practice (*n* = 132)

	Not Useful N (%)	Slightly Useful N (%)	Moderately Useful N (%)	Very Useful N (%)
Access to the internet in your workplace	11 (8.3)	3 (2.3)	22 (16.7)	**96** **(72.7)**
Access to free online databases in the workplace, (i.e., Cochrane and Pubmed)	4 (3.0)	5 (3.8)	28 (21.2)	**95** **(72.0)**
Free access to online databases that usually require license fees (i.e., DynaMed and CINAHL)	9 (6.8)	7 (5.3)	20 (15.2)	**96** **(72.7)**
Ability to download full-text/full-length journal articles	5 (3.8)	9 (6.8)	17 (12.9)	**101** **(76.5)**
Access to online education materials related to evidence-based practice	4 (3.0)	7 (5.3)	27 (20.5)	**94** **(71.2)**
Access to tools used to assist the critical appraisal/evaluation or research evidence.	3 (2.3)	16 (12.1)	36 (27.3)	**77** **(58.3)**
Access to critically appraised topics relevant to your field (these are critical appraisals of single research papers)	3 (2.3)	10 (7.6)	34 (25.8)	**85** **(64.4)**
Access to critical reviews of research evidence relevant to your field (these are critical reviews of multiple research papers addressing a single topic)	2 (1.5)	9 (6.8)	36 (27.3)	**85** **(64.4)**
Access to research rating tools that facilitate critical appraisal of single research papers	6 (4.5)	16 (12.1)	47 (35.6)	**63** **(47.7)**
Access to online tools that assist you to conduct your own critical appraisals of multiple research papers related to a single topic.	12 (9.1)	24 (18.2)	35 (26.5)	**61** **(46.2)**

Bold values indicate the highest number of responses for each item

## Discussion

The primary findings of this study were that the majority of South African chiropractors who responded to this survey are generally favourable towards EBP, and this practice therefore appears to be utilised and embraced, with the requisite skills.

### Demographics

The majority of respondents (52.3%) in our study were between the ages of 26–35 years old; we can correlate this to the 52.3% of the respondents obtaining their degree between 2010 and 2019. The American study of chiropractors had a higher age group participating in the study, with almost half of their respondents being between the ages of 50–59 and practicing for over 16 years (86%) [10]. In contrast, 31.3% of respondents in the Australian study were between 31 and 40 years old [17], which showed the diversity of respondents between different locations. Chiropractic education in South Africa has only graduated students since the late 1990’s. Specifically, the program at the University of Johannesburg was established in 2005 and would explain why the age of survey respondents falls within this range.

Almost 60% of the respondents in our study were female, which was not the case for the Australian and American studies, as males appeared to be more willing to respond (73.4% and 91%, respectively) [10, 17]. Males and females were almost evenly distributed in the research conducted on Norwegian chiropractors [18]. Our study, therefore, reflects similar trends to Yelverton et al., indicating the findings are representative of the sex demographic in South Africa as female chiropractors within the country are increasing [19]. Our findings align with global trends indicating a worldwide increase in the prevalence of female healthcare workers [20]..

In the context of our study and based on findings from the American perspective [10], it was observed that a significant proportion of chiropractors practiced independently, with solo practices accounting for 56.1% and 58.0% respectively. The preference for solo practice limits the opportunities for interaction with colleagues and hampers interprofessional collaborations [8]. Many doctors prefer solo practice because it develops more professional autonomy, which is a key principle of the medical profession [21]. However, Norwegian chiropractors preferred to work in a multidisciplinary setting with conventional healthcare providers [18]. In Australia, chiropractors were mainly situated in a suburban setting [17], similar to South Africa. The preference to practice in the city and suburban areas were demonstrated in Norway [18]. Practitioners choose to practice in urban areas because they have more time and are able to operate in a multidisciplinary environment to handle the workload [22]. These factors can have an impact on EBP attitudes and use.

### Chiropractic attitudes towards evidence-based practice

Overall, chiropractors in South Africa have a positive attitude towards EBP as they are willing to improve their skills to develop their decision-making skills to assist with patient care. By implementing EBP, patients can make informed decisions with the guidance of a chiropractor, and become educated about their condition to give them the best outcome [12]. Chiropractors across multiple countries share the same sentiments as they believe that EBP will help the profession progress and integrate into the healthcare system [8]. Science was considered more important than traditional chiropractic beliefs or philosophy in the UK [23], emphasising the need for further research to enhance chiropractic treatment. The positive outlook from South African Chiropractors regarding EBP may facilitate a unified identity and further growth of the profession within the country, especially in the management of musculoskeletal health.

### Chiropractic skills in evidence-based practice

Overall, South African chiropractors’ EBP skill range was average to above average, indicating a lack of confidence in their applied research skills. Respondents’ capacity to discover and disseminate research was adequate; however, their ability to locate relevant studies to their requirements was mediocre. In a comparable study, Americans chiropractors exhibited stronger confidence in their EBP skills except for research evidence synthesis [10]. Norwegian chiropractors displayed inadequate skills in conducting clinical research or systematic review skills [18]. These findings can be attributed to chiropractic programs that focus on locating and analysing current data rather than actively performing their own research projects, which are mostly influenced by academics in their respective fields.

### Evidence-based practice training and education

Chiropractic institutions play an important role in promoting the use of EBP [8], as respondents indicated that they received most of their knowledge on evidence-based practice from their undergraduate training. In South Africa, the chiropractic program consists of a 4-year undergraduate BHSc degree and a 2-year postgraduate MHSc Chiropractic degree, which is essential for registration and practice as a chiropractor [24, 25]. The MHSc includes a research dissertation that allows for developing skills in retrieving and applying evidence in a chiropractic setting [25]. Chiropractic orthopaedics in America report that they received most of their EBP education at a postgraduate level [10]. This is in contrast to UK and Australian chiropractors who indicated that they obtained the majority of their EBP information as undergraduates, even if it was insufficient [14, 17]. The difference in chiropractic education across countries would be determined by how the curriculum of the institutions are constructed, the emphasis placed on research, and how to use and critically analyse data from evaluated studies. Despite the fact that South Africa is similar to other countries, practitioners should be equipped with research courses and training opportunities in order to promote the use of EBP for optimal patient care.

### Chiropractic utilisation of evidence-based practice

Contrary to the Swedish study, which revealed low to moderate levels of involvement with EBP activities, approximately two-thirds of chiropractors in South Africa utilize EBP and apply their findings [26]. The use of clinical research, publications and CPGs was not the first line of information used to assist patients, nor were patient preferences taken into account when treating, which are practices that did not align with the EBP model. Engagement with EBP activities was also moderately low among Norwegian chiropractors, who consulted published research studies about 1–5 times per month on average [18]. All types of research sources were deemed valuable, however they were not used as frequently as chiropractors wanted owing to a variety of barriers. Even though South African chiropractors believe that EBP is crucial for the advancement of the profession, it does not convert into their practice as their sources are primarily personal intuition or consulting with peers.

### Chiropractic barriers and facilitators to the uptake of evidence-based practice

The findings from our study, as well as previous research, indicate that the lack of time needed to integrate EBP into a chiropractor’s practice is a significant barrier [26, 27]. The daily activities required by chiropractors do not make provisions for incorporating active research on a regular basis [8]. Most chiropractors in solo practice have numerous responsibilities to manage their business efficiently, especially when skills aren’t developed, and researching and critically appraising research may take time [21]. The lack of motivation and opportunity to search for clinically relevant research was not reported as a hindrance. Internet access, online databases, and, more specifically, open access and full-text articles were viewed as facilitators of EBP among South African chiropractors. This sentiment was shared by chiropractors in Norway and Sweden, allowing practitioners access to the most recent research for clinical practice [26, 28]. Continuing education programs for successful implementation can be recommended to overcome barriers and boost facilitators for EBP [26].

### Evidence-based practice in South Africa

Chiropractic in South Africa is still a developing profession, especially within the public healthcare system. Chiropractors recognise that research could help raise the credibility of the profession, thus further establishing interprofessional collaborations [8]. EBP has become a model that is continuously growing and setting the foundation for all healthcare professionals. There is steady advancement in aligning CPGs with EBP. In South Africa, there are no CPGs established for musculoskeletal health, which would directly influence the diagnosis and treatment plans for chiropractors. A shared knowledge repository will make it simpler for chiropractors to communicate about studies supporting various treatment techniques among peers, their patients, and other medical professionals [12] [29]. In utilising EBP on a regular basis, chiropractors can develop treatment protocols for patients to improve outcomes and overall patient satisfaction. Despite EBP being part of curriculums in chiropractic institutions, practitioners do not consistently follow through with practicing this approach. Practitioners should consider collaborating with academics at chiropractic institutions to research current issues they experience in practice, building the body of knowledge available and creating guidelines for patient management. Emphasis needs to be placed on high-quality EBP continuous professional development programs and research directed at patient-centred care to meet the needs of chiropractors in practice [1, 13, 26]. Chiropractors could consider using open-access journals to enhance clinical decision-making and to boost their research uptake by applying the practice at least daily.

### Limitations

This study, the first of its kind, offers valuable insights into the practice of South African chiropractors. However, due to the small sample size and low response rate, the findings cannot be generalised to all chiropractors in the country. Although the self-reported survey may not accurately reflect actual practices, it provides an estimation of their utilization. The self-administered survey was anonymous to reduce dishonesty and the concern of bias among respondents [30].

## Conclusions

The majority of South African chiropractors are generally favourable towards EBP, and this practice therefore appears to be utilised and embraced, with the requisite skills. A lack of clinical evidence in chiropractic and a lack of time may have contributed as barriers to the uptake of EBP.

### Electronic supplementary material

Below is the link to the electronic supplementary material.

Supplementary Material 1

## Data Availability

The datasets used and/or analysed during the current study are available from the corresponding author on reasonable request.
