# Supplementary material for: Chiropractic attitude and utilisation of evidence-based practice in South Africa: a secondary analysis
Source: Chiropr Man Therap. 2024 Apr 30;32:13. doi: 10.1186/s12998-024-00534-3 (PMC11062011; doi:10.1186/s12998-024-00534-3)
Supplement: Supplementary file 1 — Supplementary Material 1 [file 12998_2024_534_MOESM1_ESM.docx]

**EBASE Questionnaire and Scoring Rubric**

(Modified for chiropractic profession)

Thank you for agreeing to complete this important survey. Your support in completing this questionnaire is valued and greatly appreciated. The following questionnaire aims to identify the use, opinion, skills, and training in evidence-based practice (EBP) for chiropractors residing within South Africa.

This questionnaire is estimated to take approximately 10 minutes to complete and consists of 73 questions. For most questions, please indicate how you feel at the time of completing the survey by clicking the circle next to your best selection (please try to avoid answering ‘neutral’ unless you really are uncertain).

**PART A: Demographic Questions**

1. **Age:**

| 25 and younger | 1 |
| --- | --- |
| 26-35 years old | 2 |
| 36-45 years old | 3 |
| 46-55 years old | 4 |
| Other than 55 years old | 5 |

1. **Sex:**

| Male | 1 |
| --- | --- |
| Female | 2 |

1. **Chiropractic university of graduation:**

| University of Johannesburg (UJ) | 1 |
| --- | --- |
| Durban University of Technology (DUT) | 2 |
| Technikon Witwatersrand | 3 |
| Technikon Natal | 4 |
| Other (please specify):  [fillable field] | 5 |

1. **Year of Chiropractic university graduation: _______**
2. **Your highest level of education besides your MHSc/MTech degree in Chiropractic: [check one box only]**

| No other degree | 1 |
| --- | --- |
| Bachelor’s Degree | 2 |
| Honour’s Degree | 3 |
| Master’s Degree | 4 |
| Doctorate (PhD) Degree | 5 |

1. **In which province is your primary practice?**

| Gauteng | 1 |
| --- | --- |
| Kwa-Zulu Natal | 2 |
| Mpumalanga | 3 |
| Limpopo | 4 |
| North West | 5 |
| Eastern Cape | 6 |
| Northern Cape | 7 |
| Free State | 8 |
| Western Cape | 9 |

1. **In which type of geographic setting do you practice? [check one box only]**

| Urban | 1 |
| --- | --- |
| Suburban | 2 |
| Rural | 3 |

1. **What is your role in the clinical setting in which you practice? [check one box only]**

| Associate/employee | 1 |
| --- | --- |
| Sole proprietor/Solo practice | 2 |
| Sole proprietor within group | 3 |
| Owner/Partner multi-disciplinary practice | 4 |
| Partnership within group | 5 |

1. **What is the average number of patient visits that you personally (not clinic) see weekly?**

| 10 or less | 1 |
| --- | --- |
| 11-20 | 2 |
| 21-30 | 3 |
| 31-40 | 4 |
| 41-50 | 5 |
| 51-60 | 6 |
| 61-70 | 7 |
| 71-80 | 8 |
| 81-90 | 9 |
| 91-100 | 10 |
| More than 100 | 11 |

1. **What is the main focus of your chiropractic care? [check one box only]**

| Paediatrics | 1 |
| --- | --- |
| Family Care | 2 |
| Wellness/Prevention | 3 |
| Sports | 4 |
| Non-musculoskeletal care | 5 |
| Spine | 6 |
| General musculo-skeletal care (spine and extremities) | 7 |

1. **Please indicate your organizational status. [check one box only]**

| No Membership | 1 |
| --- | --- |
| CASA Member | 2 |

**PART B**

**On a scale ranging from strongly disagree to strongly agree, how would you rate your opinion on the following statements? (Please select one best answer per category)**

|  | **Strongly Disagree** | **Disagree** | **Neutral** | **Agree** | **Strongly**  **Agree** |
| --- | --- | --- | --- | --- | --- |
| **1. Evidence-based practice is necessary in the practice of chiropractic** | 1 | 2 | 3 | 4 | 5 |
| **2. Professional literature (i.e.: journals & textbooks) and research findings are useful in my day-to-day practice** | 1 | 2 | 3 | 4 | 5 |
| **3. I am interested in learning or improving the skills necessary to incorporate EBP**  **into my practice** | 1 | 2 | 3 | 4 | 5 |
| **4. Evidence-based practice improves the quality of my patient’s care** | 1 | 2 | 3 | 4 | 5 |
| **5. Evidence-based practice assists me in making decisions about patient care** | 1 | 2 | 3 | 4 | 5 |
| **6. Evidence-based practice considers my clinical experience when making clinical decisions** | 1 | 2 | 3 | 4 | 5 |
| **7. Evidence-based practice considers a patient’s preference for treatment** | 1 | 2 | 3 | 4 | 5 |
| **8. The adoption of evidence-based practice places an unreasonable demand on my practice** | 1 | 2 | 3 | 4 | 5 |

**PART C**

**On a scale from 1 to 5, with 1 being poor and 5 being advanced, how would you rate your skills in the following areas? (Please select one per skill area)**

|  | **Poor**  **1** | **2** | **3** | **4** | **Advanced**  **5** |
| --- | --- | --- | --- | --- | --- |
| **1. Identifying knowledge gaps in practice** | 1 | 2 | 3 | 4 | 5 |
| **2. Identifying answerable clinical questions** | 1 | 2 | 3 | 4 | 5 |
| **3. Locating professional literature (i.e.: journal articles)** | 1 | 2 | 3 | 4 | 5 |
| **4. Online database searching (i.e.: MEDLINE)** | 1 | 2 | 3 | 4 | 5 |
| **5. Retrieving evidence** | 1 | 2 | 3 | 4 | 5 |
| **6. Critical appraisal of evidence** | 1 | 2 | 3 | 4 | 5 |
| **7. Synthesis of research evidence** | 1 | 2 | 3 | 4 | 5 |
| **8. Applying research evidence to patient cases** | 1 | 2 | 3 | 4 | 5 |
| **9. Sharing evidence with colleagues** | 1 | 2 | 3 | 4 | 5 |
| **10. Conducting clinical research (i.e.: clinical trials)** | 1 | 2 | 3 | 4 | 5 |
| **11. Using findings from clinical research** | 1 | 2 | 3 | 4 | 5 |
| **12. Conducting systematic reviews** | 1 | 2 | 3 | 4 | 5 |
| **13. Using findings from systematic reviews** | 1 | 2 | 3 | 4 | 5 |

**PART D**

**Please indicate the highest level of training/education you have received in the following areas (please select one best answer per category). If you tick ‘other’, please write down your highest level of training/education in the space provided.**

|  | None | Major part of chiropractic education | Seminar (< 1 day) | Short course (< 1  week) | Specific course (>1  week) | Formal postgraduate training | Minor part of chiropractic education | Informal personal study (i.e., books, internet, Journals) | Other (please specify) |
| --- | --- | --- | --- | --- | --- | --- | --- | --- | --- |
| **1. Evidence-based clinical**  **practice / Evidence-based chiropractic** | 1 | 2 | 3 | 4 | 5 | 6 | 7 | 8 | 9 |
| **2. Applying research**  **evidence to clinical practice** | 1 | 2 | 3 | 4 | 5 | 6 | 7 | 8 | 9 |
| **3. Conducting clinical**  **research (i.e.: clinical trials)** | 1 | 2 | 3 | 4 | 5 | 6 | 7 | 8 | 9 |
| **4. Conducting systematic reviews or meta-analysis (i.e.: statistical analysis of data combined from two or**  **more studies)** | 1 | 2 | 3 | 4 | 5 | 6 | 7 | 8 | 9 |
| **5. Critical thinking / critical**  **analysis** | 1 | 2 | 3 | 4 | 5 | 6 | 7 | 8 | 9 |

**PART E**

**Please indicate how often you have performed the following activities over the last month (please select one best answer per category).**

|  | Never | 1-5 Times | 6-10 Times | 11-15 Times | 16+ times |
| --- | --- | --- | --- | --- | --- |
| **1. I have read/reviewed professional literature (i.e.:**  **professional journals & textbooks) related to my practice** | 1 | 2 | 3 | 4 | 5 |
| **2. I have read/reviewed clinical research findings**  **related to my practice** | 1 | 2 | 3 | 4 | 5 |
| **3. I have used professional literature or research**  **findings to assist my clinical decision-making** | 1 | 2 | 3 | 4 | 5 |
| **4. I have used professional literature or research**  **findings to change my clinical practice** | 1 | 2 | 3 | 4 | 5 |
| **5. I have used an online database (i.e.: CINAHL, MEDLINE) to search for practice related literature or research** | 1 | 2 | 3 | 4 | 5 |
| **6. I have used an online search engine (i.e.: Google) to search for practice related literature or research** | 1 | 2 | 3 | 4 | 5 |

**7. What percentage of your practice do you estimate is based on clinical research evidence (i.e.: evidence from clinical trials)? (Please tick only one response)**

| None (0%) | 1 |
| --- | --- |
| Very small proportion (1-25%) | 2 |
| Small proportion (26-50%) | 3 |
| Moderate (51-75%) | 4 |
| Large proportion (76-99%) | 5 |
| All (100%) | 6 |

**8. When you are making clinical decisions, in what order do the following sources of information inform the basis of your decision? Please rank the items from 1 to 10, with 1 being the most frequently used source of information, to 10, being the least frequently used source of information:**

Published clinical evidence (i.e.: clinical trials)

Published experimental/laboratory evidence (i.e.: animal or test tube studies)

Traditional knowledge

Consulting fellow practitioners or experts

Personal intuition

Trial and error

Textbooks

Clinical practice guidelines

Patient preference

Personal preference

**PART F**

**On a scale ranging from ‘not a barrier’ to ‘major barrier’, to what extent are the following factors barriers preventing you from participating in evidence-based practice?**

|  | Not a Barrier | Minor Barrier | Moderate Barrier | Major Barrier |
| --- | --- | --- | --- | --- |
| **1. Lack of time** | 1 | 2 | 3 | 4 |
| **2. Lack of resources (i.e.: access to a computer, the internet or online databases)** | 1 | 2 | 3 | 4 |
| **3. Lack of clinical evidence in complementary and alternative medicine** | 1 | 2 | 3 | 4 |
| **4. Insufficient skills for locating research** | 1 | 2 | 3 | 4 |
| **5. Insufficient skills for interpreting research** | 1 | 2 | 3 | 4 |
| **6. Insufficient skills to critically appraise / evaluate the literature** | 1 | 2 | 3 | 4 |
| **7. Insufficient skills to apply research findings to clinical practice** | 1 | 2 | 3 | 4 |
| **8. Lack of incentive to participate in evidence-based practice** | 1 | 2 | 3 | 4 |
| **9. Lack of interest in evidence-based practice** | 1 | 2 | 3 | 4 |
| **10. Lack of relevance to chiropractic practice** | 1 | 2 | 3 | 4 |
| **11. Lack of university support for evidence- based practice** | 1 | 2 | 3 | 4 |
| **12. Lack of industry support for evidence-based practice** | 1 | 2 | 3 | 4 |
| **13. Patient preference for treatment** | 1 | 2 | 3 | 4 |

**PART G**

**On a scale ranging from ‘not useful’ to ‘very useful’, to what extent would the following strategies assist you in participating in evidence-based practice?**

|  | Not Useful | Slightly Useful | Moderately Useful | Very Useful |
| --- | --- | --- | --- | --- |
| **1. Access to the Internet in your workplace** | 1 | 2 | 3 | 4 |
| **2. Access to free online databases in the**  **workplace, such as Cochrane and PubMed** | 1 | 2 | 3 | 4 |
| **3. Free access to online databases that usually require license fees, such as**  **DynaMed and CINAHL** | 1 | 2 | 3 | 4 |
| **4. Ability to download full text / full-length**  **journal articles** | 1 | 2 | 3 | 4 |
| **5. Access to online education materials**  **related to evidence-based practice** 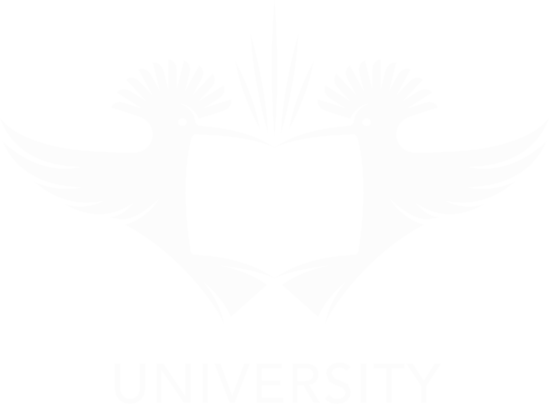 | 1 | 2 | 3 | 4 |
| **6. Access to tools used to assist the critical**  **appraisal / evaluation of research evidence** | 1 | 2 | 3 | 4 |
| **7. Access to critically appraised topics relevant to your field (these are critical**  **appraisals of single research papers)** | 1 | 2 | 3 | 4 |
| **8. Access to critical reviews of research evidence relevant to your field (these are critical reviews of multiple research papers**  **addressing a single topic)** | 1 | 2 | 3 | 4 |
| **9. Access to research rating tools that**  **facilitate critical appraisal of single research papers** | 1 | 2 | 3 | 4 |
| **10. Access to online tools that assist you to conduct your own critical appraisals**  **of multiple research papers related to a**  **single topic** | 1 | 2 | 3 | 4 |

**RUBRIC FOR CALCULATING EBASE SUB-SCORES**

**PART B: Attitude Sub-score**

**Summary of part B**

- Number of items: 8 items
- Response type: 5-point Likert scale (strongly disagree to strongly agree)
- Score per item: 1 (strongly disagree) to 5 (strongly agree)
- Range of possible total scores: 8-40

**Scoring**

1. Score items #1 (B1) through to #8 (B8) as outlined above
2. Reverse Score B8
3. Sum all scores for items #1 (B1) through to #8 (B8)
4. Scores can be interpreted using the following quartiles:
5. Q1=08-16 (predominantly strongly disagree to disagree)
6. Q2=17-24 (predominantly neutral to disagree)
7. Q3=25-31 (predominantly neutral to agree)
8. Q4=32-40 (predominantly agree to strongly agree)

**PART C: Skill Sub-score**

**Summary of part C**

- Number of items: 13 items
- Response type: 5-point skill scale (poor skill to advanced skill)
- Score per item: 1 (poor skill) to 5 (advanced skill)
- Range of possible total scores: 13-65

**Scoring**

1. Score items #1 (C1) through to #13 (C13) as outlined above
2. Sum all scores for items #1 (C1) through to #13 (C13)
3. Scores can be interpreted using the following quartiles:
4. Q1=13-26 (predominantly poor to somewhat poor)
5. Q2=27-39 (predominantly somewhat poor to average)
6. Q3=40-51 (predominantly average to somewhat advanced)
7. Q4=52-65 (predominantly somewhat advanced to advanced)

**PART D: Training Sub-score**

**Summary of part D**

- Number of items: 5 items
- Response type: 9-point training scale (no training to other training)
- Score per item: 1 (no training) to 9 (other training)
- Range of possible total scores: 9-45

**Scoring**

1. Score items #1 (D1) through to #5 (D5) as outlined above
2. Sum all scores for items #1 (D5) through to #5 (D5)

**PART E: Use Sub-score**

**Summary of part E**

- Number of items: 6 items
- Response type: 5-point use scale (never to 16+ times)
- Score per item: 1 (never) to 5 (16+ times)
- Range of possible total scores: 6-30

**Scoring**

1. Score items #1 (E1) through to #6 (E6) as outlined above
2. Sum all scores for items #1 (E1) through to #6 (E6)

**PART F: Barrier Sub-score**

**Summary of part F**

- Number of items: 13 items
- Response type: 4-point skill scale (no barrier to major barrier)
- Score per item: 1 (no barrier) to 4 (major barrier)
- Range of possible total scores: 4-52

**Scoring**

1. Score items #1 (F1) through to #13 (F13) as outlined above
2. Sum all scores for items #1 (F1) through to #13 (F13)

**PART G: Useful Strategies Sub-score**

**Summary of part G**

- Number of items: 10 items
- Response type: 4-point skill scale (not useful to very useful)
- Score per item: 1 (not useful) to 4 (very useful)
- Range of possible total scores: 4-40

**Scoring**

1. Score items #1 (G1) through to #10 (G10) as outlined above
2. Sum all scores for items #1 (G1) through to #10 (G10)
